# Current challenges and opportunities in the care of patients with fibrodysplasia ossificans progressiva (FOP): an international, multi-stakeholder perspective

**DOI:** 10.1186/s13023-022-02224-w

**Published:** 2022-04-18

**Authors:** Robert J. Pignolo, Christopher Bedford-Gay, Amanda Cali, Michelle Davis, Patricia L. R. Delai, Kristi Gonzales, Candace Hixson, Alastair Kent, Hope Newport, Manuel Robert, Christiaan Scott, Frederick S. Kaplan

**Affiliations:** 1 Department of Medicine, Mayo Clinic, Rochester, MN USA; 2 International FOP Association, Kansas City, MO USA; 3 FOP Friends, Manchester, UK; 4 The Radiant Hope Foundation, Mountain Lakes, NJ USA; 5 The Ian Cali FOP Research Fund, PENN Medicine, Center for Research in FOP & Related Disorders, Philadelphia, PA USA; 6 Tin Soldiers: Global Patient Identification Program, Johannesburg, South Africa; 7 Hospital Israelita Albert Einstein, Instituto de Ensino E Pesquisa, Sao Paolo, Brazil; 8 Independent Patient Advocacy Consultant, Downham Market, UK; 9 Fundación FOP, Buenos Aires, Argentina; 10 Patient Author, Buenos Aires, Argentina; 11 Red Cross Children’s Hospital, University of Cape Town, Cape Town, South Africa; 12 Departments of Orthopaedic Surgery and Medicine, The Center for Research in FOP and Related Disorders, Perelman School of Medicine, University of Pennsylvania, Philadelphia, PA USA

**Keywords:** Fibrodysplasia ossificans progressiva, FOP, Unmet needs, Patient care, Diagnosis, Health policy, Rare disease

## Abstract

**Background:**

Fibrodysplasia ossificans progressiva (FOP) is an ultra-rare, disabling genetic disorder characterized by congenital malformations of the great toes and progressive heterotopic ossification of soft and connective tissues. Assiduous attention to the unmet needs of this patient community is crucial to prevent potential iatrogenic harm and optimize care for individuals with FOP.

**Objective:**

To gather international expert opinion and real-world experience on the key challenges for individuals with FOP and their families, highlight critical gaps in care, communication, and research, and provide recommendations for improvement.

**Methods:**

An international group of expert clinicians, patients and patient advocates, caregivers and representatives from the international FOP community participated in a virtual, half-day meeting on 22 March 2021 to discuss the key unmet needs of individuals with FOP.

**Results:**

Individuals with FOP often face the frustration of long diagnostic journeys, the burden of self-advocacy and the navigation of novel care pathways. Globally, patients with FOP are also confronted with inequities in access to diagnosis and specialist care, and consequently, unequal access to registries, clinical trials, and essential support from patient associations. Organizations such as the International FOP Association, the International Clinical Council on FOP, and national FOP organizations work to provide information, facilitate access to expert clinical guidance, nurture patient empowerment, fund FOP research and/or foster meaningful collaborations with the research community. The non-profit Tin Soldiers Global FOP Patient Search program aims to identify and provide a pathway to diagnosis and care for individuals with FOP, particularly in underserved communities. Such global initiatives and the increasingly widespread use of telemedicine and digital platforms offer opportunities to improve vital access to care and research.

**Conclusions:**

This multi-stakeholder perspective highlights some of the unmet needs of individuals with FOP and their families. Regional and international organizations play an important role in improving the quality of life of those they reach in the global FOP community. However, globally, fundamental issues remain around raising awareness of FOP among healthcare professionals, identifying individuals with FOP, reducing time to diagnosis, and ensuring access to best practice in care, support, and clinical research. Medical writing support was industry-sponsored.

**Supplementary Information:**

The online version contains supplementary material available at 10.1186/s13023-022-02224-w.

## Introduction

Fibrodysplasia ossificans progressiva (FOP; OMIM #135100) is an ultra-rare, disabling genetic disorder characterized by congenital malformations of the great toes and progressive heterotopic ossification (HO) of soft and connective tissues [1]. Approximately 97% of individuals with FOP carry the same spontaneous missense mutation in the *ALK2/ACVR1* gene, involved in the bone morphogenetic protein (BMP) signaling pathway [2, 3]. The estimated prevalence of FOP (diagnosed cases) has been reported as up to 1.43 per million individuals [4]; however, regional variability is high [4, 5].

Episodes of soft tissue swelling, pain, reduced movement, stiffness and warmth, referred to as ‘flare‐ups’, often emerge in young children with FOP [6]. Although FOP flare‐ups can occur spontaneously, they are frequently triggered by intra-muscular injections, unnecessary biopsies, muscle fatigue, dental work, minor trauma or influenza‐like viral illnesses [7]. Flare-ups often lead to HO [8], but FOP progression can also occur in the absence of flare-ups [6]. HO is cumulative and permanent, leading to progressive disability and severe functional limitations in joint mobility. As individuals age, their need for assistance in performing activities of daily living increases and, by the third decade of life, most individuals with FOP are confined to a wheelchair [9]. Many clinical aspects of FOP resemble a premature aging phenotype [10].

Individuals with FOP have a median life expectancy of 56 years [11]. However, a wide variation in lifespan has been observed, with some patients reaching their seventies [11]. In underserved regions,^1^ the effect of limited access to diagnosis and care on the life expectancy of individuals living with FOP remains unknown. Death is often a result of cardiorespiratory failure, due to thoracic insufficiency syndrome, or pneumonia [11]. There is no definitive medical treatment for FOP; current clinical management is supportive and aims to mitigate the risks that could cause further disease progression and complications [12]. Attempts to treat individuals with FOP by physicians who lack the necessary experience or knowledge of FOP can inadvertently result in flare-ups and/or irreversible disease progression.

Due to the rarity and disabling nature of FOP, patients and their caregivers require specialist, multi-disciplinary care, and support with many aspects of life. The FOP community faces numerous challenges including low awareness of FOP among healthcare professionals (HCPs) and policymakers, high rates of misdiagnosis worldwide [13], poor access to specialist FOP care, and limited support and information for individuals who are isolated from the global FOP community [4]. These factors are exacerbated in developed or developing regions where access to healthcare is limited due to resource constraints and/or competing healthcare challenges, such as high rates of communicable diseases. This includes highly populated regions such as Asia and Africa, where rates of FOP diagnosis are largely unknown but most likely very low. The recent increase in targeted clinical trials offers hope that an effective disease-modifying therapy, or therapies, may be identified [14]. However, such developments also present new medical and logistical challenges for individuals with FOP, and it is vital to implement provisions to ensure equitable access to care, support, and research. Identifying the most important challenges and raising awareness of these among policymakers is crucial for improving quality of life for individuals with FOP and their families worldwide.

A multi-stakeholder meeting was convened to give a voice to the FOP community, represented by an international group of clinical experts/researchers from the International Clinical Council on FOP (ICC) [15], patients, patient advocates, caregivers, and members of FOP organizations such as the International FOP Association (IFOPA) [16], FOP Friends [17], Tin Soldiers [18] and Fundación FOP [19]. Drawing on their collective expertise and/or lived experience, the multi-stakeholder group highlighted the main challenges for patients with FOP and their caregivers, priority areas for change, and opportunities to further improve the care of people living with FOP worldwide. This paper presents the outcomes of these discussions along with recommendations for the achievement of best practice and global equity in FOP care.

## Methods

A targeted literature review was conducted to identify recent evidence on awareness of FOP, unmet needs and best practices in FOP care, and patient engagement with research. The information gathered was used to develop a proposed agenda of discussion topics for the multi-stakeholder meeting.

An international group of expert clinicians (representing the ICC), patient representatives/advocates, caregivers, and representatives from the IFOPA, Tin Soldiers, and other FOP national organizations, were invited to participate in a virtual, half-day meeting to provide a broad range of perspectives on the key unmet needs of individuals with FOP and their families. A project charter outlining the roles and responsibilities of all parties involved in the project was shared with prospective attendees ahead of the meeting, for full transparency. Consistent with best-practice reporting of patient involvement in research, we report patient involvement in this project using the standardized Guidance for Reporting Involvement of Patients and the Public (GRIPP) checklist (Additional file 1: Table S1) [20].

**Fig. 1:**
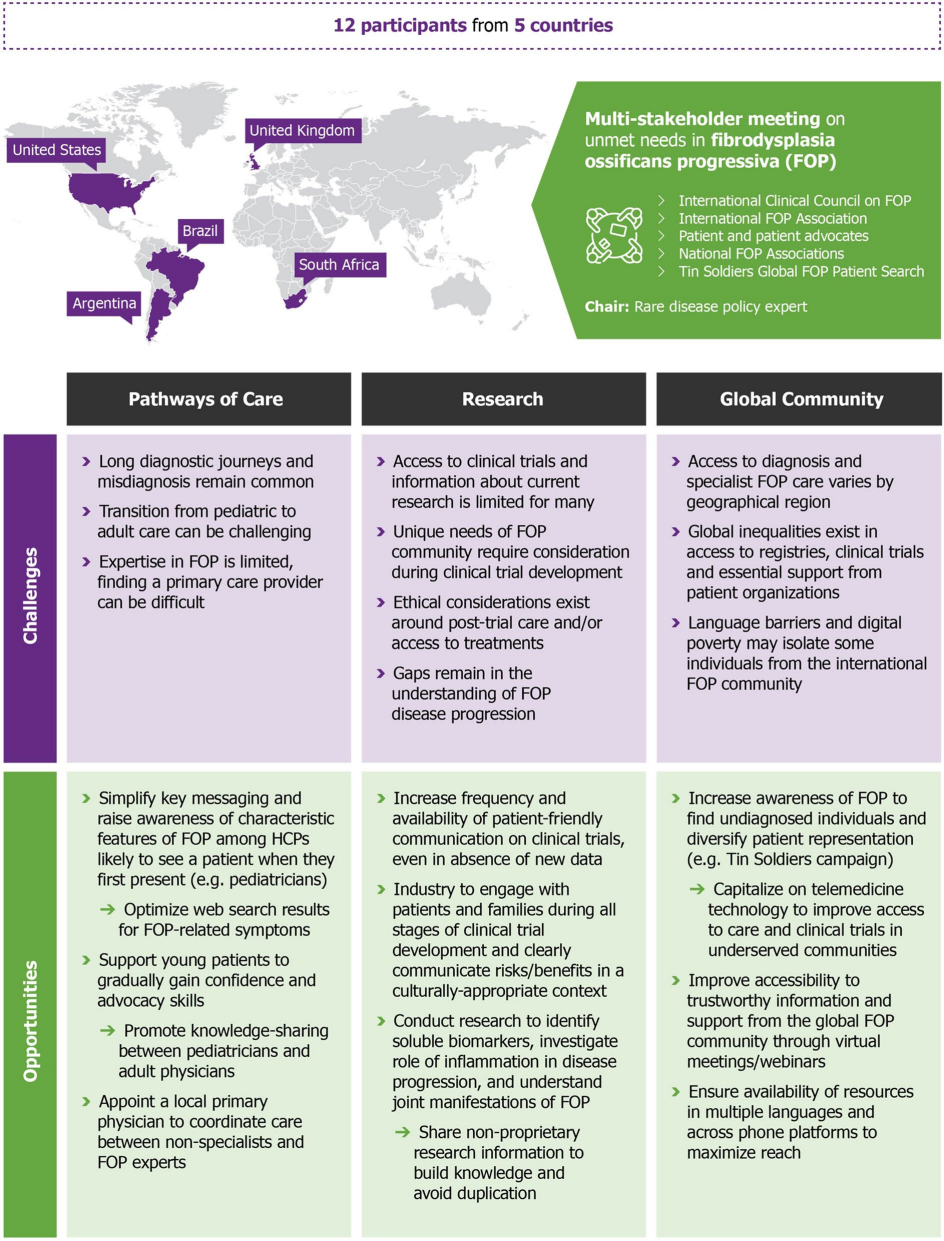
Overview of the multi-stakeholder meeting and key challenges and opportunities identified. A multi-stakeholder meeting was convened to give a voice to the FOP community, represented by an international group of clinical experts/researchers from the ICC, patients, patient advocates, and caregivers, representing FOP Friends, Tin Soldiers and Fundación FOP, and members of the IFOPA. Drawing on their collective expertise and/or lived experience, the multi-stakeholder group highlighted the main challenges for patients with FOP and their caregivers, priority areas for change, and opportunities to further improve the care of people living with FOP worldwide. FOP: fibrodysplasia ossificans progressiva; HCP: healthcare professional; ICC: International Clinical Council on FOP; IFOPA: International FOP Association

A total of 12 participants attended the virtual meeting, held on 22 March 2021. The multi-stakeholder group included individuals from 5 countries (Argentina, Brazil, South Africa, United Kingdom and the United States of America). An expert in rare disease policy development and patient engagement was selected to chair the meeting. The format of the meeting was organized as a virtual roundtable discussion, with a participant introducing each discussion topic with a short talk, followed by open discussion. Topics were developed around pathways of care, patient involvement in research, and future opportunities made possible by advancements in technology. Discussions held both during the meeting and the development of the manuscript were analyzed thematically and resulted in the identification of key challenges and opportunities for improving the care of individuals with FOP worldwide (Fig. 1).

## Pathways to care

### Diagnostic journey for patients and families

Delayed diagnosis is a common phenomenon across rare diseases [21]. Although time to diagnosis for individuals with FOP has decreased in recent years [13, 22], patients and their caregivers still face a prolonged diagnostic journey. Data from the FOP Registry (as of December 2020) show that on average, individuals with FOP receive a correct diagnosis around 1.5 years following symptom onset, with over 50% of individuals initially misdiagnosed [13, 22]. Common incorrect diagnoses include cancer (e.g. osteosarcoma), juvenile fibromatosis and non-hereditary myositis ossificans [13, 23]. Prolonged diagnostic journeys and misdiagnosis may be more common for individuals with an atypical FOP mutation [22] and/or for those who live in an underserved region. As a result of misdiagnosis, it is common for patients with FOP to undergo unnecessary surgical procedures or medical interventions. Such interventions can exacerbate disease progression and contribute to disability [23]. Therefore, the timely and accurate diagnosis of FOP is crucial to prevent premature loss of mobility and preserve quality of life for individuals with FOP [23].

According to recent FOP Registry data, individuals with FOP receive a correct diagnosis after seeing an average of 3.3 HCPs [13]. These results highlight that there is a disconnect between the HCPs first consulted and those who are most likely to accurately diagnose patients [9, 13]. Geneticists and orthopedic physicians were identified as the specialists most likely to provide a definitive diagnosis of FOP [9, 13, 22]. Although newborn screening for FOP has recently been proposed in Brazil, the implementation of these programs more widely is, in part, limited by country-specific differences in newborn screening policies. However, there are also important ethical considerations over screening for a disease with no approved treatment, concerns over the risk of misdiagnosis based on clinical observation in newborns, and regulatory/cost-effectiveness barriers related to genetic testing. Therefore, the routine screening of newborns to diagnose FOP, either clinically or genetically, is unlikely to become more widespread in the near future. Efforts should focus on raising awareness of the characteristic features of FOP among the HCPs who are most likely to see patients early in life or when they first present with symptoms.

Diagnosis of FOP can be made clinically. Individuals will often present in their first decade of life with great toe malformations and lumps/swellings on the scalp, head, neck and/or back [6]; FOP is the only condition with this combination of symptoms. Increased communication of this unique symptom combination among pediatricians and other HCPs may help to reduce the frequency of misdiagnosis and shorten the time to a correct diagnosis and care. Consideration of these symptoms in isolation, particularly the lumps and swellings characteristic of FOP, can lead to misdiagnosis and unnecessary medical procedures, potentially causing irreparable harm. The ability to provide a clinical diagnosis of FOP is particularly valuable for individuals living in regions where genetic testing is either not available or cost prohibitive. However, where available, genetic testing can also be beneficial, particularly for individuals with an atypical genetic mutation or for those who have great toe malformations without HO [24].

There has been a recent drive to optimize rare disease diagnosis through the use of web search, social media and medical data repositories in recognition that many parents, caregivers, and HCPs increasingly use these resources to search for a diagnosis [25]. Although such approaches can be helpful, it is important that individuals are steered towards trustworthy sources of information and advice. The IFOPA is currently optimizing search results to ensure that individuals who search for FOP-related symptoms, such as malformations of the great toes, are directed to the IFOPA website and the wide variety of resources provided (Table 1) [16]. Increasing the accessibility of reliable information available online for both patients and HCPs could help to reduce patient/caregiver anxiety and the time to diagnosis.

**Table 1:** Online resources available for individuals with FOP

Resources	Details
IFOPA [16]	
Medical care resources	Personalized medical brochure; medical information tracking resources; emergency medical cards
Resources for those newly diagnosed	IFOPA Welcome Packet; FOP Connect: Peer Mentor Program
Ability Toolbox Program	Designed to empower individuals with FOP by promoting independence through the use of tools and home adaptations
Resilient Living Program	Provides education for strengthening the mind and spirit as every family works to overcome the challenges they face throughout their journey with FOP
The Advocacy Series	Provides the tools for individuals to become more effective communicators and problem solvers in a variety of settings
Educational videos, webinars and workshops available on YouTube channel	Including: ‘The Same but Different: A Look at Life with FOP’; FOP Facts and Insights; Oral Health and Anesthesia workshop; ‘FOP and COVID-19’
Support guidebooks	‘What is FOP? A Guidebook for Families’; ‘What is FOP? Questions and Answers for the Children’
Resources for children with FOP and their families	Including: Guidance for siblings; ‘Supporting your Child During a Stressful Procedure’; ‘Summer Family Activity Guide’; ‘FOP and School’; ‘Kids with FOP and Sports’; Fun Zone at FOP Family Gathering; tween/teen journaling workshop
Meetings	FOP Family Gathering
Clinical studies and trials guidance	Glossary of terms; current list of ongoing clinical studies and trials; videos and webinars such as ‘Development Journey for FOP: How Medicines Become Treatments’ and ‘Exploring Clinical Trials for FOP’
ICC [15]	
FOP Treatment Guidelines	Includes ‘Executive Summary of Key Practice Points’ [12]
Clinical trial guidance	Current list of ongoing clinical trials; tips on how to prepare for clinical trials; publication on ‘Special considerations for clinical trials in fibrodysplasia ossificans progressiva (FOP)’ [47]
COVID-19 guidance	Precautions for FOP Patients and Families [60]; Vaccine recommendations [61]
FOP Friends [17]	
Supporting a Child with FOP: a practical guide to their learning experience	Written by experienced teachers, to offer clear information and advice for how to best support a child with FOP and their family; serves as an ongoing guide providing useful age-appropriate insights and considerations as the child ages

COVID-19: coronavirus disease; FOP: fibrodysplasia ossificans progressiva; ICC: International Clinical Council on FOP; IFOPA: International FOP Association

A recent study of the epidemiology of FOP, based on information from the patient registration databases of the IFOPA and 16 other regional/national FOP organizations, found that the apparent prevalence of individuals with FOP ranged from approximately 0.65 per million in North America to 0.05 and 0.04 per million in Africa and the Asia Pacific region, respectively [4]. The authors concluded that this variability is likely associated with limited awareness of FOP, delays in diagnosis, lack of supporting regional infrastructure, and poor access to a local FOP organization or the international FOP community in underserved areas [4]. To address global inequalities in access to diagnosis, the Tin Soldiers Global FOP Patient Search program aims to increase global awareness of FOP, to identify undiagnosed individuals and connect patients with the FOP community [18]. By employing a novel approach that used a mixed-media campaign and community-based programs, the Tin Soldiers initiative increased the number of known cases in Africa from 25 to 32 over a five-month period from December 2020 to April 2021 [26]. These newly diagnosed individuals were subsequently connected to national and international support structures, patient organizations and medical care. This positive outcome demonstrates the potential of a dedicated global patient identification program to increase rates of diagnosis and access to care pathways for individuals living in underserved regions.

### Education for healthcare professionals

There is a need for more unified, global initiatives to educate HCPs on caring for patients with rare bone diseases. Many scientific societies are expanding the educational programming available at congresses and using special journal issues to raise awareness, highlight recent research, and present new perspectives on rare bone diseases [27]. To address the need to educate HCPs on caring for patients with FOP specifically, the ICC created the Global Health & Education Task Force with the following goals: (1) identify FOP global health providers; (2) develop a global database of treating FOP physicians, across all relevant specialties; (3) formulate a pathway for new ICC membership; and (4) develop education platforms for new members.

Many countries are also becoming increasingly aware of the importance of providing educational programs for HCPs to develop their expertise in rare diseases. These programs can be used to encourage collaboration and the sharing of information among HCPs (locally and globally) and also to foster communication between HCPs and patient organizations [28]. For example, the Rare Bone Disease TeleECHO, a monthly video teleconference, promotes active learning and peer-to-peer knowledge sharing of rare bone diseases for HCPs around the world [29]. Similarly, the Tin Soldiers Continuing Medical Education (CME) Master series provides virtual and international educational opportunities for HCPs to interact with and learn about FOP from their peers [30]. However, in areas of digital poverty other initiatives may be needed, such as enlisting the help of local faith-based health initiatives and/or humanitarian medical non-governmental organizations to reach physicians and nurses [18].

Although it may not be feasible to educate all HCPs on FOP-specific symptoms, simplifying the key messaging for clinicians around rare diseases may expedite the diagnosis process for all individuals with a rare disease. For example, a drive to raise awareness among clinicians that the presence of a bilateral physical malformation is possibly indicative of a genetic disease, may increase the likelihood of timely referral to a geneticist. Messaging specifically targeted to the pediatric healthcare community (e.g. pediatricians and pediatric nurses, nurse practitioners and physician assistants, and the neonatal care team) and/or the most common frontline HCPs in a given country (i.e. community pharmacists in Egypt, Jordan, Lebanon and Somalia [31]) is also needed to improve the likelihood of an individual receiving a correct diagnosis prior to receiving any unnecessary and/or potentially harmful medical procedures.

### Care coordination

As FOP does not fall under one medical specialty, patients with FOP interact with a number of HCPs and specialists during the management of their care. A coordinated, multidisciplinary approach to providing care for patients with FOP is critical to ensure high-quality care and patient safety [24, 32]. However, expertise in rare diseases is often concentrated in a limited number of medical centers, typically where clinicians have taken a special interest in the rare disease [21]. This is also the case for FOP, where the standard of care and access to specialists for individuals with FOP can vary considerably by geographic region.

**Fig. 2:**
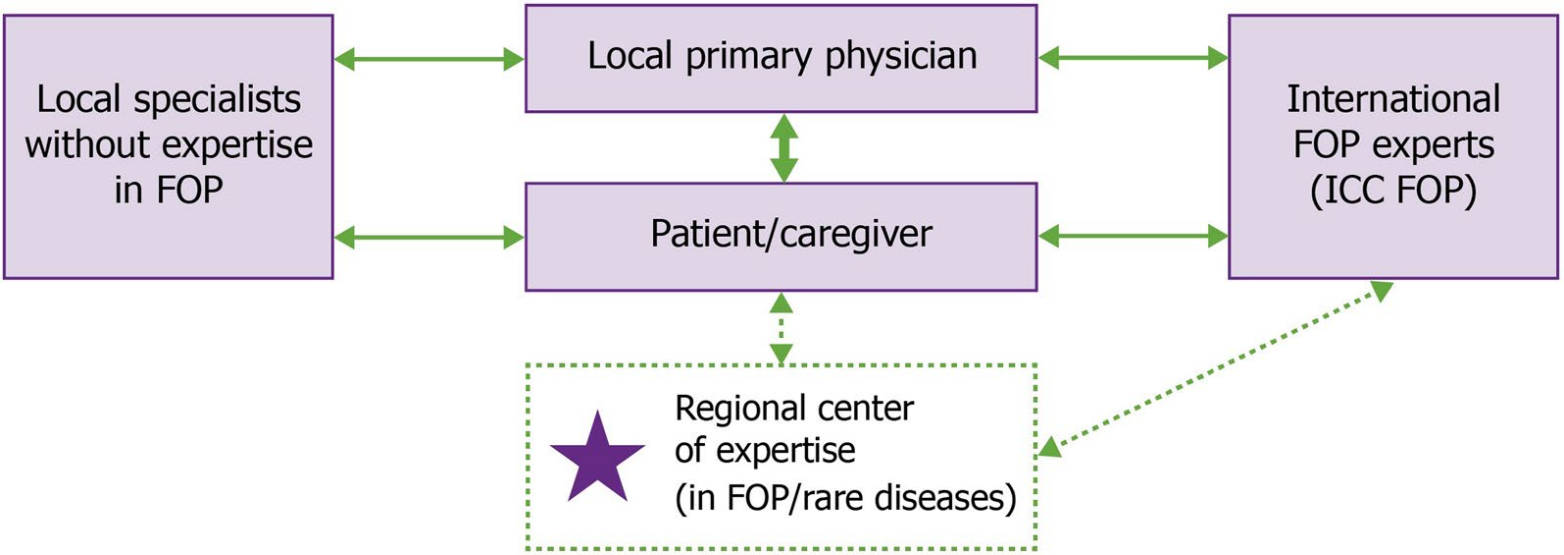
Care coordination pathway (current and future). The journey to diagnosis for patients with FOP can involve a number of different HCPs. The IFOPA and other national/global FOP organizations and initiatives also play an important role in the identification and referral of patients. Following diagnosis, patients with FOP should be supported by a primary physician who is willing to consult with FOP experts and can coordinate a local care team of specialists without expertise in FOP [12]. FOP experts from the ICC can provide guidance and education for the local physician as required. There are also a variety of CME opportunities available for all HCPs. As the global network of FOP specialists continues to expand, it is the long-term goal of the ICC to create multiple national/regional centers of FOP care. These centers would combine medical, surgical, anesthesia, physical and occupational therapy, and dental expertise in FOP in physical locations to improve care and minimize risks for patients. These centers could also function as key sites for clinical research. CME: Continuing Medical Education; FOP: fibrodysplasia ossificans progressiva; HCP: healthcare professional; ICC: International Clinical Council on FOP; IFOPA: International FOP Association

With variable access to specialists, patients should have a primary physician who can coordinate a local care team and who is willing to consult with FOP experts [12] (Fig. 2). With the help of the patient community and national organizations, the ICC is actively recruiting and continually expanding its global network of FOP specialists through the Global Health & Education Task Force [15]. In addition, the Tin Soldiers formed the ‘African Clinicians Collective’ that includes ten African clinicians, nine of whom currently treat individuals with FOP [18]. Nevertheless, despite best efforts, it is not feasible for FOP experts to be involved in every care decision. To assist local HCPs with the day-to-day management of patients with FOP, the ICC maintains up-to-date FOP Treatment Guidelines, last updated in April 2021 [12]. These guidelines provide an important resource for HCPs, but are also invaluable for patients and/or their caregivers to advocate for themselves in a medical setting.

There is an increased awareness that specialized treatment, knowledge, and resources for the care management of rare and complex diseases need to be centralized. For example, European Reference Networks (ERNs) were launched in 2017 as virtual networks of HCPs across Europe to facilitate discussion and improve the management of rare diseases [33]. Specifically, the RarERN Path has been developed as a reference organizational model for care pathways that can be adapted to best fit the specific disease and geographical context [33]. The long-term goal of the ICC’s global network of FOP specialists is the development of national/regional centers of FOP care; combining medical, surgical, anesthesia, physical and occupational therapy, and dental expertise in FOP in physical locations to improve care and minimize risks for patients [24]. In addition to focusing on care, these centers could also function as key sites for clinical research. Many existing centers currently serve as clinical trial sites for ongoing interventional studies.

### Transition from pediatric to adult care

The transition from pediatric to adult care, also known as transitional care, has been characterized as “the purposeful, planned movement of adolescents and young adults with chronic physical and medical conditions from child-centered to adult-oriented health care systems” [34]. For chronic health conditions, the transition from pediatric to adult care can be challenging for the patient and caregiver [35], and this is especially true for rare diseases [36]. For the patient, assuming responsibility for managing insurance, prescriptions, diet, dental care, and other key needs can be overwhelming, regardless of the specific underlying disease. The importance of a successful transition to adult care for health outcomes has been established [35, 36]. Individuals who encounter difficulties navigating the transition to adult care can often experience disruptions in care that increase the likelihood of future medical complications, higher emergency department/hospital use, lower treatment adherence and reduced quality of life [37]. Similar to other rare diseases [36], individuals with FOP often experience a delayed and disrupted transition to adult care.

The transition from familiar physicians, family-centered systems, and multidisciplinary pediatric care to a less supportive and more fragmented adult healthcare system can be a particularly challenging time for patients and their caregivers [36]. One cause of anxiety for patients with a rare disease is that adult physicians often lack the necessary expertise and experience of treating patients with rare diseases, especially those that are first encountered in childhood [36]. There is often a related fear among patients and their caregivers that adult physicians may not consider or value the patient’s own expertise and lived experience [37, 38]. As an ultra-rare disease that does not fall under one medical specialty, it is particularly challenging to manage adult care for individuals with FOP. It can be difficult to find a primary physician willing to assume responsibility for the coordination of a patient’s care into adulthood. Identifying an adult-care physician early on in the transition process and including them in care management discussions with the patient, caregivers and pediatrician can help to mitigate the anxiety experienced by patients and caregivers, and ensure the continuity of high-quality, specialized care [36–38].

Active involvement of the patient and caregivers in care decision-making has been shown to increase the likelihood of a successful transition to adult care [36, 39]. To facilitate anticipatory care, and to account for variability in FOP disease progression, it is beneficial to start preparing an individual for the transition to adult care from an early age [37]. Although learning self-advocacy skills can be overwhelming at first, with the support of a guardian, a young patient can gradually gain experience and confidence in assuming an active role in their own care management. It may also be beneficial for individuals who share an FOP diagnosis to help support and mentor younger individuals and their families during the transition [40]. The goal should be to ensure connectivity and continuity of care, in an age- and disease progression-appropriate context. The IFOPA provides many resources and programs to help patients emotionally and practically negotiate this transition, including the Transition of Care webinars and the Advocacy Series and Resilient Living Program (Table 1). In addition, national FOP organizations play a key role in providing targeted support and guidance for families within local communities.

### Patient empowerment and support

At present, patients or caregivers typically assume primary responsibility for informing and educating new clinicians, dentists, or other care providers about FOP and how to manage care for individuals with FOP. However, developing advocacy skills can be time-consuming and challenging, and not every patient or caregiver has the ability to act as an advocate. As the life expectancy for individuals with FOP increases, it may become difficult for aging caregivers to provide the level of physical care needed (e.g. lifting) over time. Therefore, it is important to educate and empower a patient’s wider support network, such as their adult siblings, and not just their primary caregiver.

The IFOPA and other national organizations specifically aim to educate and nurture advocacy skills to empower patients and their support network by providing many online resources for managing day-to-day and emergency care (Table 1; Additional file 1: Table S2). An important aspect of managing day-to-day care, particularly for children with FOP, is within an educational setting. Therefore, organizations such as the IFOPA and FOP Friends have created resources in a child-friendly format to help raise awareness of FOP among educators and school-age children to ensure the safety of children with FOP among their peers (Table 1). In addition to physical care, providing emotional support is a fundamental but often overlooked and underprioritized aspect of care [24], and the IFOPA has created resources to address the social and mental health needs of the FOP community. This support can be particularly important for individuals who did not develop symptoms nor receive a diagnosis of FOP until their teenage years, after experiencing a relatively normal childhood.

Social media and mobile phone messaging apps are very important to the FOP community and serve as a platform to ask questions, share resources, feel connected to peers, and problem-solve in real time. In addition to providing practical information and guidance for parents of children with a rare disease, access to support groups and communication with other parents is an important social need [41]. Communities created through online and mobile phone platforms and supported by patient organizations can encourage a network of collaborative self-management among patients and caregivers that can enhance care and lead to better clinical outcomes [21]. Social media can also be an important tool for children with FOP to interact with their peers and mitigate isolation.

However, it is important to recognize the limitations of these tools. Social media can be difficult to navigate for individuals with underlying mental health issues, such as anxiety or depression. Furthermore, richer interactions may be required to offer optimal support. Although efforts have been made to expand reach across various digital/mobile phone platforms and languages, maintaining clear messaging across platforms can be challenging. Additional barriers can be caused by limited access to the internet and enabled devices in some parts of the world [42]. Nevertheless, these tools offer the opportunity to increase access and vital connectivity to regional or international FOP communities for individuals worldwide.

## Research

### Patient involvement and access

The identification of the classic FOP gene mutation (*ALK2/ACVR1*^R206H^) and the dysregulated BMP signaling pathway was a key milestone in the history of FOP [2]. The discovery identified druggable targets for treatments and fueled international interest in research and development (R&D) in FOP [2, 14]. This recent drive in R&D has resulted in several promising treatments for FOP reaching clinical trial stage [43]. Patients played a pivotal role in the discovery of the FOP gene and continue to be instrumental in advancing FOP research by being actively involved in online surveys and participating in advisory panels for industry, clinical trials, non-interventional studies, the “Tooth Ferry” Program, and the IFOPA’s FOP Registry and FOP Biobank [44–46].

Patients are often keen to participate in clinical trials, but opportunities can be limited (particularly in the Global South). Pharmaceutical companies often rely on existing patient networks, created and maintained through a patient organization or specialist clinicians, to increase awareness of an upcoming trial and to identify participants. The lack of an established, local patient network can, therefore, be a major barrier to initiating a clinical trial in a certain geographical region. Other barriers to establishing clinical trials in underserved areas include cumbersome country-specific legal regulations that cause delays or roadblocks, a very low number of known diagnosed patients, lack of local supportive infrastructure for clinical trials and inadequate research training, and prioritization of local sites for trials of treatments for more prevalent infectious diseases.

As there is an increased drive to expand clinical trials into new regions and increase participation for underserved patient groups, there are important ethical concerns to be considered. It is crucial that the relevant risks and benefits of a clinical trial are clearly communicated, taking into account cultural context; the message should convey that clinical trials are experiments, not proven treatments [14, 47]. There are also ethical considerations around access to post-trial care and/or treatments for participants following a clinical trial. This is especially important if the treatment provided during the trial resulted in a meaningful clinical benefit for the participant [48].

Guidance for clinical trials in FOP have recently been developed by the Clinical Trials Committee of the ICC [47]. These guidelines emphasize the responsibility of pharmaceutical companies to engage with patients and their families during all stages of clinical trial development. The patient perspective should be reflected in the practicalities of the trial (e.g. need for onsite visits, imaging, etc.), but also to provide insight into the FOP-specific safety considerations that are necessary to include in the trial design, and the assessment of measurable outcomes that are meaningful to patients [47]. Ensuring that the voice of the patient is heard and incorporated into research studies and clinical trial design is essential to maintaining a positive relationship between the FOP community and the pharmaceutical industry.

Maintaining patient-friendly communication with the FOP community throughout a clinical trial or research study is important, even when there is no new information to provide. The IFOPA provides educational resources to explain the drug development process and how clinical studies and trials are conducted to enable individuals to accurately interpret research results and understand how these findings may be relevant to themselves or their family members. Although there are many possible avenues to communicate research outcomes, some of the most accessible and well-known to the FOP community are the Annual Reports of the FOP Collaborative Research Project at the University of Pennsylvania [46], lay summaries provided by IFOPA for research projects that they fund, and the IFOPA’s FOP Family Gathering. In addition, the FOP Drug Development Forum organized by the IFOPA brings together academic researchers, clinical care specialists and patients to discuss and plan future research with the patient voice central to these discussions. However, there is a need for all stakeholders to go beyond the proactive communication of research to actively sharing non-proprietary research information with the FOP community to build knowledge and limit the duplication of research. As such, the IFOPA, in collaboration with the ICC, has developed guidelines for data-sharing specific to various research activities. This “Open Science” approach to data sharing and collaboration has been identified as particularly important within the rare disease community to reduce the time to diagnosis and treatment [49].

National patient organizations and networks also play an important role in communicating research updates and information to individuals within a given country or region (Additional file 1: Table S2). For example, the national FOP organization for Argentina (Fundación FOP) connects the Spanish-speaking patients and families throughout Latin America with the international FOP community. However, there are still regions that are not directly supported by a patient organization, which can be very isolating for patients and their families [4].

To improve access to care, reliable information, and clinical studies and trials for all individuals with FOP, there have been several recent initiatives to increase regional representation and expand the reach of the international FOP community. For example, the FOP Registry, launched in 2015 by the IFOPA, is a centralized, international registry for patients with FOP that captures demographic data and has facilitated longitudinal studies of patient health and quality of life [50]. As of December 2020, the Registry had 323 enrolled patients from 69 countries, approximately 36% of the world’s known FOP population. In addition to providing valuable research information, it is hoped that the Registry will increase the known patient base for this ultra-rare disease and improve clinical care by increasing the speed of development of novel treatments [44]. It is important to note that language (the Registry is currently available in seven languages), educational level and/or limited access to the internet/enabled devices may be barriers to participation in the FOP Registry for some individuals. Addressing these issues that limit regional representation and ethnic diversity are crucial to ensure that patients, regardless of their location, receive appropriate care and treatment, access to clinical trials and up-to-date information and support.

### Gaps in research

Despite many crucial advancements arising from FOP research from over 60 institutions worldwide, such as the discovery of the genetic mutation underlying FOP [2], there is still much to understand about disease progression. The natural course and co-morbidities of FOP in poorly resourced areas remain particularly understudied. Future research should also prioritize the identification of biomarkers that can be used to predict flare-up and HO status, and evaluate treatment efficacy. In addition, as FOP shares similarities with other inflammatory diseases, understanding the role of inflammation in the context of FOP could provide valuable insight and new avenues to treatments. Finally, although FOP is often categorized as a “new bone” disease, it is also a disease of the joints [51, 52]. As such, further research on the joint manifestations of FOP is warranted.

Natural history studies of FOP are important to understand disease progression and to identify clinically meaningful outcome measures to assess in short-term (typically 1–2 years) clinical trials [6]. However, conducting these studies in ultra-rare diseases can be fraught with challenges, including small numbers of geographically-dispersed patients and a lack of established biomarkers, validated measurement tools, and clinically-meaningful disease progression endpoints. A recent 3-year, global natural history study of FOP (NCT02322255; sponsored by Ipsen) provided valuable information on the baseline, cross-sectional disease phenotypes of 114 individuals with FOP [52–54] and additional longitudinal data are forthcoming. These studies will be crucial to better understand the natural history of FOP and identify common secondary health issues. In addition, the increased use of FOP-specific assessments, such as the Cumulative Analogue Joint Involvement Scale (CAIJS), will help to better understand the disease progression of FOP [55]. An initial clinical staging system for FOP has also been proposed [56], which is currently being validated using data from a longitudinal natural history study on FOP and the FOP Registry. Further expansion of the FOP Registry will continue to yield valuable data on the longitudinal natural history of FOP for patients globally, and can also be used in the future to facilitate post-marketing surveillance of new therapies/treatments [50].

## Future directions

Changes to ‘routine’ medical practice and the rise of telemedicine during the coronavirus disease (COVID-19) pandemic could provide a framework to improve access to care and clinical studies/trials for individuals with FOP around the world. Telemedicine can facilitate access to specialists for the management of care in FOP, particularly for patients who are not able to travel or have limited access to local medical specialists.

By necessity, remote medical assessments became the norm during the COVID-19 pandemic. As the feasibility and benefits of remote medical consultations are recognized and understood, this may lead to positive changes regarding how data are collected for patient registries, disease diagnosis, natural history studies and clinical trials, ultimately improving accessibility [49]. Wearable medical devices transmitting digital health data may also be a way to increase access to research and clinical trials for individuals [49]. For example, FOP-PROMPT is a patient-reported outcomes (PRO) tool that can be used to capture the signs, symptoms and impacts most important to people living with FOP via a mobile phone app [57]. It may be possible to incorporate this daily symptom tracker into future clinical trials to enhance the timespan of data collected. However, not all medical information can be collected remotely (e.g. whole-body computed-tomography scans) and the importance of in-person interaction between a patient and clinician cannot be minimized [49].

Although the COVID-19 pandemic has been an isolating time for patients and their families, the circumstances necessitated by the pandemic have forced a new level of comfort with video-conferencing and online webinars. The IFOPA has seen increased engagement with online meetings of the international FOP community, such as the annual FOP Family Gathering. This event is an opportunity for families and local HCPs to learn about the latest developments in treatments and care from FOP experts, and tips and strategies for disease management and daily living from other people living with FOP. Traditionally an in-person event, the necessitated virtual format increased accessibility to individuals who would have been unable to travel and participate in person. Digital platforms can provide an opportunity for individuals to connect with the international FOP community while efforts are ongoing to establish dedicated patient organizations in underserved countries and regions. The events organized by national and regional patient organizations, whether in person or virtual, are crucial to build the FOP community and provide support and information for individuals (Table 1) [4].

Although there are many opportunities for digital platforms and telemedicine to have a positive impact on the global FOP community in the future, it is also important to be aware of limitations posed by cost, digital poverty, and language barriers. For example, although the 2020 IFOPA Virtual FOP Family Gathering was translated into 17 languages, it was not possible to provide translation for every language due to the associated costs. Individuals also have differential access to the internet and data based on their location and means, including rural communities in developed countries [58]. To reach patients who do not have access to the internet, national telephone helplines can be used to reach a higher number of individuals in their local language at limited cost [59]. Similarly, it may be possible to make key information available on a mobile phone app that can be referred to during medical appointments.

## Conclusions

As an ultra-rare, disabling disease with no definitive treatment at present, FOP poses significant challenges for patients and their caregivers. Individuals with FOP often face a long diagnostic journey and a constant need to advocate for themselves in new medical circumstances. Continued education of HCPs on the characteristics of FOP, particularly targeting those HCPs most likely to see a patient when they first develop symptoms, will help to reduce the rate of misdiagnosis and shorten the diagnostic journey for patients. Once a diagnosis is received, a local primary physician should be appointed to coordinate care between local non-specialists and FOP experts. Furthermore, as the transition from pediatric to adult care can be challenging for individuals and often result in disruptions to care, it is important to identify a primary adult physician early to encourage knowledge-sharing with the pediatrician and ensure a positive transition process.

With ongoing and anticipated clinical trials, there is hope that an effective disease-modifying therapy, or therapies, will be identified. However, there are safety and ethical concerns to consider during clinical trial development that relate to the unique needs of the FOP community, post-trial care and/or access to new treatments. The patient perspective should be reflected at all stages of clinical trial development to ensure that FOP-specific safety considerations are addressed and that the measurable outcomes are meaningful to patients. Communications on FOP research should be frequent and conveyed in plain language that is easily translatable, to ensure the timely sharing of information globally and reduce the spread of misinformation or misunderstanding due to poor translation. Although additional research is needed to fully understand the disease progression of FOP, the sharing of non-proprietary research information between stakeholders will help to further build knowledge and limit duplication.

In many regions of the world, individuals have poor access to diagnosis and expert care, face barriers to participating in registries and trials, and may be isolated from the wider FOP community. Innovations in telemedicine and increased communication via digital platforms have the potential to improve accessibility of care and information worldwide. It is critical to ensure that all individuals with FOP are afforded equitable access to care and treatment. Along with the continuing efforts from the IFOPA, the ICC, national FOP organizations, the Tin Soldiers Global FOP Patient Search, and Industry, increasing awareness of the unmet needs facing the FOP community among key audiences will be vital to address these challenges and improve the care of patients globally.

## Supplementary Information

**Additional file 1: Table S1.** GRIPP2 patient and public involvement. **Table S2.** National FOP patient organizations.

## Data Availability

Not applicable.

## Footnotes

1 In the context of this paper, the term ‘underserved’ is used to indicate any community, whether in a developed or developing region, that faces inequities in access to information, diagnosis, specialist care and/or support from patient organizations.

## References

[1] Kaplan FS, Tabas JA, Gannon FH, Finkel G, Hahn GV, Zasloff MA (1993). The histopathology of fibrodysplasia ossificans progressive. An endochondral process. J Bone Joint Surg Am.

[2] Shore EM, Xu M, Feldman GJ, Fenstermacher DA, Cho TJ, Choi IH (2006). A recurrent mutation in the BMP type I receptor ACVR1 causes inherited and sporadic fibrodysplasia ossificans progressiva. Nat Genet.

[3] Zhang W, Zhang K, Song L, Pang J, Ma H, Shore EM (2013). The phenotype and genotype of fibrodysplasia ossificans progressiva in China: a report of 72 cases. Bone.

[4] Liljesthröm M, Pignolo R, Kaplan F (2020). Epidemiology of the global fibrodysplasia ossificans progressiva (FOP) community. J Rare Dis Res Treat.

[5] Baujat G, Choquet R, Bouée S, Jeanbat V, Courouve L, Ruel A (2017). Prevalence of fibrodysplasia ossificans progressiva (FOP) in France: an estimate based on a record linkage of two national databases. Orphanet J Rare Dis.

[6] Pignolo RJ, Bedford-Gay C, Liljesthröm M, Durbin-Johnson BP, Shore EM, Rocke DM (2016). The natural history of flare-ups in fibrodysplasia ossificans progressiva (FOP): a comprehensive global assessment. J Bone Miner Res.

[7] Kaplan FS, Pignolo RJ, Al Mukaddam M, Shore EM, Bilezikian J (2019). Genetic Disorders of Heterotopic Ossification: Fibrodysplasia Ossificans Progressiva and Progressive Osseous Heteroplasia. Primer on the metabolic bone diseases and disorders of mineral metabolism.

[8] Pignolo RJ, Shore EM, Kaplan FS (2011). Fibrodysplasia ossificans progressiva: clinical and genetic aspects. Orphanet J Rare Dis.

[9] Pignolo RJ, Cheung K, Kile S, Fitzpatrick MA, De Cunto C, Al Mukaddam M (2020). Self-reported baseline phenotypes from the International Fibrodysplasia Ossificans Progressiva (FOP) Association Global Registry. Bone.

[10] Pignolo RJ, Wang H, Kaplan FS. Fibrodysplasia ossificans progressiva (FOP): a segmental progeroid syndrome. Front Endocrinol. 2020;10(908). 10.3389/fendo.2019.00908 PMC6966325 31998237

[11] Kaplan FS, Zasloff MA, Kitterman JA, Shore EM, Hong CC, Rocke DM (2010). Early mortality and cardiorespiratory failure in patients with fibrodysplasia ossificans progressiva. J Bone Joint Surg Am.

[12] Kaplan FS, Al Mukaddam M, Baujat G, Brown M, Cali A, Cho T-J (2021). The medical management of fibrodysplasia ossificans progressiva: current treatment considerations. Proc Int Clin Council FOP.

[13] Sherman LA, Cheung K, De Cunto C, Kile S, Pignolo RJ, Kaplan FS. The diagnostic journey in fibrodysplasia ossificans progressiva: insights from the FOP registry. Annual Meeting of the American Society for Bone and Mineral Research. 2020.

[14] Kaplan F, Al Mukaddam M, Baujat G, Cali A. The Twilight Zone: Benefit, Risk & Hope in Clinical Trials for Fibrodysplasia Ossificans Progressiva (FOP). The ICC. 2020.

[15] International Clinical Council on Fibrodysplasia Ossificans Progressiva. https://www.iccfop.org/. Accessed 29 April 2021.

[16] International Fibrodysplasia Ossificans Progressiva Association. https://www.ifopa.org/. Accessed 29 April 2021.

[17] FOP Friends. https://fopfriends.com/. Accessed 19 May 2021.

[18] Tin Soldiers Global FOP Patient Search. https://tinsoldiers.org/. Accessed 19 May 2021.

[19] Fundación FOP. http://fundacionfop.org.ar/. Accessed 14 July 2021.

[20] Staniszewska S, Brett J, Simera I, Seers K, Mockford C, Goodlad S (2017). GRIPP2 reporting checklists: tools to improve reporting of patient and public involvement in research. BMJ.

[21] Stoller JK (2018). The challenge of rare diseases. Chest.

[22] International Fibrodysplasia Ossificans Progressiva Association (IFOPA). 2020 FOP Registry Annual Report. 2021. https://www.ifopa.org/2020_fop_registry_annual_report. Accessed 18 May 2021.

[23] Kitterman JA, Kantanie S, Rocke DM, Kaplan FS (2005). Iatrogenic harm caused by diagnostic errors in fibrodysplasia ossificans progressiva. Pediatrics.

[24] Di Rocco M, Baujat G, Bertamino M, Brown M, De Cunto CL, Delai PLR (2017). International physician survey on management of FOP: a modified Delphi study. Orphanet J Rare Dis.

[25] Svenstrup D, Jørgensen HL, Winther O (2015). Rare disease diagnosis: a review of web search, social media and large-scale data-mining approaches. Rare Diseases.

[26] Scott C, Kaplan F, Friedman C, Delai P, Al Mukaddam M, Cali A, et al. While looking for one, you may find another: Tin Soldiers and the search for undiagnosed individuals with fibrodysplasia ossificans progressiva (FOP). Pediatr Rheumatol. 2021:19(Suppl 1):O6.

[27] Shore EM, Pacifici M (2019). JBMRPlus: Special Issue on Rare Bone Diseases 2019. JBMR plus.

[28] Dharssi S, Wong-Rieger D, Harold M, Terry S (2017). Review of 11 national policies for rare diseases in the context of key patient needs. Orphanet J Rare Dis.

[29] Tosi LL, Rajah EN, Stewart MH, Gillies AP, Hart TS, Lewiecki EM (2020). The rare bone disease TeleECHO program: leveraging telehealth to improve rare bone disease care. Curr Osteoporos Rep.

[30] Tin Soldiers Fibrodysplasia Ossificans Progressiva (FOP) Global CME Master Series. https://tinsoldiers.org/master_series/. Accessed 19 May 2021.

[31] El Bizri L, Jarrar LG, Ali WKA, Omar AH (2021). The role of community pharmacists in increasing access and use of self-care interventions for sexual and reproductive health in the Eastern Mediterranean Region: examples from Egypt, Jordan, Lebanon and Somalia. Health Res Pol Syst.

[32] Kilmartin E, Grunwald Z, Kaplan FS, Nussbaum BL (2014). General anesthesia for dental procedures in patients with fibrodysplasia ossificans progressiva: a review of 42 cases in 30 patients. Anesth Analg.

[33] Talarico R, Cannizzo S, Lorenzoni V, Marinello D, Palla I, Pirri S, et al. RarERN Path: a methodology towards the optimisation of patients’ care pathways in rare and complex diseases developed within the European Reference Networks. Research Square; 2020. 10.1186/s13023-020-01631-1 PMC7734838 33317578

[34] Blum RWM, Garell D, Hodgman CH, Jorissen TW, Okinow NA, Orr DP (1993). Transition from child-centered to adult health-care systems for adolescents with chronic conditions: a position paper of the Society for Adolescent Medicine. J Adolesc Health.

[35] Schraeder K, Dimitropoulos G, McBrien K, Li JY, Samuel S (2020). Perspectives from primary health care providers on their roles for supporting adolescents and young adults transitioning from pediatric services. BMC Fam Pract.

[36] Stepien KM, Kieć-Wilk B, Lampe C, Tangeraas T, Cefalo G, Belmatoug N (2021). Challenges in transition from childhood to adulthood care in rare metabolic diseases: results from the first multi-center European survey. Front Med (Lausanne).

[37] White PH, Cooley WC (2018). Supporting the health care transition from adolescence to adulthood in the medical home. Pediatrics.

[38] Rutishauser C, Akré C, Surìs JC (2011). Transition from pediatric to adult health care: expectations of adolescents with chronic disorders and their parents. Eur J Pediatr.

[39] Coyne I, Sheehan A, Heery E, While AE (2019). Healthcare transition for adolescents and young adults with long-term conditions: qualitative study of patients, parents and healthcare professionals’ experiences. J Clin Nurs.

[40] Doyle M (2015). Peer support and mentorship in a US rare disease community: findings from the cystinosis in emerging adulthood study. Patient.

[41] Pelentsov LJ, Laws TA, Esterman AJ (2015). The supportive care needs of parents caring for a child with a rare disease: a scoping review. Disabil Health J.

[42] Hootsuite & We Are Social. Digital 2021 April Global Statshot Report. 2021. https://blog.hootsuite.com/simon-kemp-social-media/. Accessed 17 June 2021.

[43] Pignolo RJ, Kaplan FS (2020). Druggable targets, clinical trial design and proposed pharmacological management in fibrodysplasia ossificans progressiva. Expert Opin Orphan Drugs.

[44] FOP Registry. Available from: https://www.ifopa.org/fopregistry. Accessed 5 May 2021.

[45] IFOPA FOP Biobank. Available from: https://www.ifopa.org/biobank. Accessed 5 May 2021.

[46] Kaplan FS, Al Mukaddam MM, Shore EM. 27th Annual Report of the Fibrodysplasia Ossificans Progressiva (FOP) Collaborative Research Project. Perelman School of Medicine at the University of Pennsylvania; 2019.

[47] Hsiao EC, Di Rocco M, Cali A, Zasloff M, Al Mukaddam M, Pignolo RJ (2019). Special considerations for clinical trials in fibrodysplasia ossificans progressiva (FOP). Br J Clin Pharmacol.

[48] Cho HL, Danis M, Grady C (2018). Post-trial responsibilities beyond post-trial access. Lancet.

[49] Rubinstein YR, Robinson PN, Gahl WA, Avillach P, Baynam G, Cederroth H (2020). The case for open science: rare diseases. JAMIA Open.

[50] Mantick N, Bachman E, Baujat G, Brown M, Collins O, De Cunto C (2018). The FOP connection registry: design of an international patient-sponsored registry for fibrodysplasia ossificans progressiva. Bone.

[51] Towler OW, Peck SH, Kaplan FS, Shore EM (2021). Dysregulated BMP signaling through ACVR1 impairs digit joint development in fibrodysplasia ossificans progressiva (FOP). Dev Biol.

[52] Towler OW, Shore EM, Kaplan FS (2020). Skeletal malformations and developmental arthropathy in individuals who have fibrodysplasia ossificans progressiva. Bone.

[53] Pignolo RJ, Baujat G, Brown MA, De Cunto C, Di Rocco M, Hsiao EC (2019). Natural history of fibrodysplasia ossificans progressiva: cross-sectional analysis of annotated baseline phenotypes. Orphanet J Rare Dis.

[54] Kou S, De Cunto C, Baujat G, Wentworth KL, Grogan DR, Brown MA (2020). Patients with ACVR1R206H mutations have an increased prevalence of cardiac conduction abnormalities on electrocardiogram in a natural history study of Fibrodysplasia Ossificans Progressiva. Orphanet J Rare Dis.

[55] Kaplan FS, Al Mukaddam M, Pignolo RJ (2017). A cumulative analogue joint involvement scale (CAJIS) for fibrodysplasia ossificans progressiva (FOP). Bone.

[56] Pignolo RJ, Kaplan FS (2018). Clinical staging of fibrodysplasia ossificans progressiva (FOP). Bone.

[57] International Fibrodysplasia Ossificans Progressiva Association (IFOPA). FOP-PROMPT Development Study Underway. https://www.ifopa.org/fop_prompt_development_study_underway. Accessed 13 July 2021.

[58] Crowe AL, McKnight AJ, McAneney H (2019). Communication needs for individuals with rare diseases within and around the healthcare system of Northern Ireland. Front Public Health.

[59] Mazzucato M, Houyez F, Facchin P (2014). The importance of helplines in National Plans. Orphanet J Rare Dis.

[60] International Clinical Council on Fibrodysplasia Ossificans Progressiva (ICC). Coronavirus (COVID-19) Precautions for FOP Patients & Families. 2020. http://www.iccfop.org/dvlp/wp-content/uploads/2020/12/Coronavirus_Precautions_for_FOP_Families_123020.pdf. Accessed 19 May 2021.

[61] International Clinical Council on Fibrodysplasia Ossificans Progressiva (ICC). Coronavirus (COVID-19) Vaccine recommendations. 2021. http://www.iccfop.org/dvlp/wp-content/uploads/2021/03/Coronavirus-vaccination-FOP-031821-1.pdf. Accessed 19 May 2021.

