# Supplementary material for: Current challenges and opportunities in the care of patients with fibrodysplasia ossificans progressiva (FOP): an international, multi-stakeholder perspective
Source: Orphanet J Rare Dis. 2022 Apr 18;17:168. doi: 10.1186/s13023-022-02224-w (PMC9014788; doi:10.1186/s13023-022-02224-w)
Supplement: Supplementary file 1 — Additional file 1: Table S1. GRIPP2 patient and public involvement. Table S2. National FOP patient organizations. [file 13023_2022_2224_MOESM1_ESM.docx]

**SUPPLEMENTARY INFORMATION**

**Supplementary Table S1.** GRIPP2 patient and public involvement

| **Section and Topic** | **Item** |
| --- | --- |
| 1: Aim | - To convene a multi-stakeholder meeting, including patients and caregivers, to identify and discuss key challenges, priority areas for change, and opportunities to improve care for people living with fibrodysplasia ossificans progressiva (FOP) - To develop a shared-voice publication based on these discussions with meeting participants as authors, and to make recommendations for improvement |
| 2: Methods | - Two patients and four patient advocates/caregivers were invited to participate (in collaboration with the International FOP Association); one of the two invited patients did not participate in the project - A project charter was developed and shared with all meeting participants/authors at the start of the project to clearly outline the project objectives and all parties’ roles and responsibilities - The patient/caregivers were integral to the discussions during the meeting, which formed the framework and basis for the paper, commented and added to the minutes from the meeting, reviewed and critically appraised multiple drafts of the manuscript [and approved the final version for submission] |
| 3: Study results | - **Positive:** During the meeting, their ‘real-world’ insight based on personal experience was fundamental to identifying the key challenges and opportunities for people living with FOP; representing three countries, they also helped to ensure that topics were considered from an international perspective. Their feedback on the minutes, and manuscript drafts were honest, insightful, and incredibly valuable. - Participants found it valuable to be part of a multi-stakeholder group and to exchange different perspectives on the content of the manuscript. Participants also found it rewarding to gain experience of authoring a peer-reviewed publication and contribute to the FOP community in a new way. - **Learning point:** For those individuals with limited experience of manuscript development, it would have been beneficial to provide a dedicated, informal session to discuss the publication process in detail and provide a space to answer any questions or concerns separate to the participants with experience of the publication process. |
| 4: Discussion and conclusions | - The patient/caregivers validated that this project would address an important unmet need for the FOP community; this project would not have gone ahead without their participation. Their input shaped the discussions in the meeting and the manuscript itself. - The ‘landscape of FOP’ is complex and constantly changing, requiring continuous, timely, and honest input from all relevant stakeholders. Perspectives from patients and patient advocates/carers are crucial to this collective discussion. |
| 5: Reflections/critical perspective | - Assistance from the International FOP Association was invaluable to identify and invite patients and caregivers who may be interested in participating in this endeavour. The virtual format of the meeting meant that participants from around the world could attend without needing to travel, which in many instances would have been prohibitive. |

The GRIPP2 is a standardized checklist used to report patient and public involvement in health and social care research ([20](file:///H:\journals\Springer\SpACE\13023\2224\author\FOP%20Policy%20Paper_Submission_04Aug21.docx#_ENREF_20)). FOP: fibrodysplasia ossificans progressiva; GRIPP: Guidance for Reporting Involvement of Patients and the Public.

**Supplementary Table S2.** National FOP patient organizations

| **Country/region with national patient organization** | **Name of patient organization** |
| --- | --- |
| Argentina | Fundación FOP |
| Australia | FOP Australia |
| Brazil | FOP Brasil |
| Canada | Canadian FOP Network (CFOPN) |
| China | - |
| Denmark | **Svenska FOP-föreningen** |
| Finland | **Svenska FOP-föreningen** |
| France | FOP France |
| Germany | FOP eV |
| India | FOP India |
| Italy | FOP Italia Onlus |
| Mexico | FOP Mexico |
| The Netherlands | FOP Stichting Nederland |
| Norway | **Svenska FOP-föreningen** |
| Poland | FOP Polska |
| Russia | FOP Russia |
| South Africa/continent of Africa | FOP Africa |
| South Korea (Republic of Korea) | **Korean FOP Overcome Family (KFOPOF)** |
| Spain | Asociación Española de Fibrodisplasia Osificante Progresiva (AEFOP) |
| Sweden | **Svenska FOP-föreningen** |
| United Kingdom | **FOP Friends^®^** |
| United States | International FOP Association (IFOPA) |

FOP: fibrodysplasia ossificans progressiva.
